# Evaluating a Remotely Delivered Cardio-Oncology Rehabilitation Intervention for Patients With Breast Cancer (REMOTE-COR-B): Protocol for a Single-Arm Feasibility Trial

**DOI:** 10.2196/53301

**Published:** 2024-04-05

**Authors:** Camille E Short, Jonathan C Rawstorn, Tamara L Jones, Lara Edbrooke, Sandra C Hayes, Ralph Maddison, Sophie Nightingale, Hilmy Ismail, Richard De Boer, Fiona Hegi-Johnson, Aaron L Sverdlov, Robyn Bell, Irene Halligan, Linda Denehy

**Affiliations:** 1 Melbourne Centre for Behaviour Change Melbourne School of Psychological Sciences The University of Melbourne Melbourne Australia; 2 Department of Physiotherapy Melbourne School of Health Sciences The University of Melbourne Melbourne Australia; 3 Institute for Physical Activity and Nutrition Deakin University Geelong Australia; 4 Department of Health Services Research The Peter MacCallum Cancer Centre Melbourne Australia; 5 Cancer Council Queensland Brisbane Australia; 6 Department of Surgical Oncology The Peter MacCallum Cancer Centre Melbourne Australia; 7 Department of Anaesthesia, Pain and Perioperative Medicine The Peter MacCallum Cancer Centre Melbourne Australia; 8 Department of Radiation Oncology The Peter MacCallum Cancer Centre Melbourne Australia; 9 Sir Peter MacCallum Department of Oncology The University of Melbourne Melbourne Australia; 10 Newcastle Centre of Excellence in Cardio-Oncology The University of Newcastle, Hunter Medical Research Institute, Calvary Mater Newcastle Newcastle Australia; 11 College of Health, Medicine and Wellbeing The University of Newcastle Newcastle Australia; 12 Consumer representative Melbourne Australia

**Keywords:** breast cancer survivor, breast cancer, cancer survivor, cancer, cardiac rehabilitation, cardiac, cardiotoxicity, cardiovascular disease, digital health, efficacy, exercise, exercise, feasibility, fitness, rehabilitation intervention, rehabilitation, safety

## Abstract

**Background:**

Exercise rehabilitation is a promising strategy for reducing cardiovascular disease risk among patients with breast cancer. However, the evidence is primarily derived from programs based at exercise centers with in-person supervised delivery. Conversely, most patients report a preference for home-based rehabilitation. As such, there is a clear need to explore strategies that can provide real-time supervision and coaching while addressing consumer preferences. Evidence from cardiac rehabilitation has demonstrated the noninferiority of a smartphone-based telerehabilitation approach (REMOTE-CR) to improve cardiorespiratory fitness in people with cardiovascular disease compared to a center-based program.

**Objective:**

This study aims to assess the feasibility, safety, and preliminary efficacy of the REMOTE-CR program adapted for patients with breast cancer at risk of cardiotoxicity (REMOTE-COR-B). We will also assess the satisfaction and usability of REMOTE-COR-B.

**Methods:**

We will conduct a single-arm feasibility study of the REMOTE-COR-B program among patients with stage I-III breast cancer who are at risk of cardiotoxicity (taking treatment type and dose, as well as other common cardiovascular disease risk factors into account) and who are within 24 months of completing primary definitive treatment. Participants (target sample size of 40) will receive an 8-week smartphone-based telerehabilitation exercise program involving remotely delivered real-time supervision and behavior change support. The platform comprises a smartphone and wearable heart rate monitor, as well as a custom-built smartphone app and web application. Participants will be able to attend remotely monitored exercise sessions during set operating hours each week, scheduled in both the morning and evening. Adherence is the primary outcome of the trial, assessed through the number of remotely monitored exercise sessions attended compared to the trial target (ie, 3 sessions per week). Secondary outcomes include additional trial feasibility indicators (eg, recruitment and retention), safety, satisfaction, and usability, and objective and patient-reported efficacy outcomes (cardiovascular fitness, quality of life, fatigue, self-reported exercise, self-efficacy, habit strength, and motivation). Adherence, feasibility, and safety outcomes will be assessed during the intervention period; intervention satisfaction and usability will be assessed post intervention; and objective and patient-reported efficacy outcomes will be assessed at baseline, post intervention (2-month postbaseline assessment), and at follow-up (5-month postbaseline assessment).

**Results:**

Recruitment for this trial commenced in March 2023, and 7 participants had been recruited as of the submission of the manuscript. The estimated completion date for the project is October 2024, with results expected to be published in mid-2025.

**Conclusions:**

The REMOTE-COR-B intervention is a novel and promising approach to providing exercise therapy to patients with breast cancer at risk of cardiotoxicity who have unique needs and heightened safety risks. This project will provide important information on the extent to which this approach is satisfactory to patients with breast cancer, safe, and potentially effective, which is necessary before larger-scale research or clinical projects.

**Trial Registration:**

Australian New Zealand Clinical Trials Registry ACTRN12621001557820; www.anzctr.org.au/ACTRN12621001557820.aspx

**International Registered Report Identifier (IRRID):**

DERR1-10.2196/53301

## Introduction

With increased rates of breast cancer survival, especially beyond 5 years, there is now an increased need to focus on the late adverse effects of cancer treatment [1,2]. The adverse impact of cancer treatment on cardiovascular health is one such late effect [3]. Cardiovascular disease (a group of heart and blood vessel disorders [4]) is now a leading cause of death among survivors of breast cancer, and survivors of breast cancer have a higher risk compared to the general population [3,5]. In addition to the overlapping risk factors of cancer and cardiovascular disease, including obesity and physical inactivity [6], the cardiotoxic nature of common breast cancer treatments (including chemotherapy, radiotherapy, and human epidermal growth factor receptor [HER]–targeted therapy) is a contributing factor to this increased risk [3]. Anthracycline-based chemotherapy and trastuzumab are of particular concern, being associated with a 5-fold increase in the risk of heart failure compared to treatment without these agents [7].

Exercise rehabilitation focused on increasing cardiorespiratory fitness is a promising strategy for reducing cardiovascular disease risk among survivors of breast cancer [8]. Lower fitness is associated with an increased risk of cardiovascular disease and all-cause mortality in the general population [9], and fitness declines have been observed during cancer treatment. Following breast cancer, major cardiovascular events tend to emerge initially around 4 years after adjuvant treatments, with a second peak around 10 years post treatment [10]. A recent study in over 4000 women, 12 years after a diagnosis of breast cancer, found that increased physical activity (equivalent to approximately 3 hours of brisk walking per week or ≥9 metabolic equivalent task hours [MET-hrs]) was associated with a 56% reduced risk of cardiovascular events (including heart failure, myocardial infarction, angina, coronary revascularization, peripheral arterial disease, carotid artery disease, transient ischemic attack, stroke, and cardiovascular death) when compared to women who exercised less (<9 MET-hrs per week) [11]. To date, over a dozen randomized controlled trials have demonstrated that exercise rehabilitation can effectively increase cardiorespiratory fitness among survivors of breast cancer [8]. Alongside consistent evidence from randomized controlled trials that exercise also improves quality of life and physical functioning [12], this evidence has led to exercise being recommended in guidelines as a routine part of cancer management [13,14].

While the evidence for exercise rehabilitation improving fitness among survivors of breast cancer is strong, it has primarily been derived from center-based exercise rehabilitation programs, where participants receive real-time in-person supervision. However, reliance on center-based delivery is likely to limit accessibility and uptake [12], as many survivors of breast cancer report a preference for home-based rehabilitation [15]. Even with this preference, it is important to ensure participant safety as well as the suitability and individualization of exercise prescriptions. Particularly because exercise trials typically recruit more “well” survivors of cancer, who are generally younger, less likely to be obese, and who are more physically active (ie, rarely include those most at-risk of cardiovascular disease) [16]. As such, there is a clear need to explore noncenter-based delivery models that can provide real-time supervision and coaching to optimize safety, particularly for high-risk patients with breast cancer (eg, those at high risk of cardiovascular disease). Evidence from the cardiac rehabilitation setting suggests that the use of sensors and mobile technologies is a promising strategy for reducing cardiovascular disease risk among survivors of breast cancer, warranting investigation [17,18].

Maddison et al [19] developed a smartphone-based exercise telerehabilitation program (REMOTE-CR) that allowed participants to receive real-time remote exercise supervision and coaching from an exercise professional. A noninferiority, randomized trial (n=162) compared REMOTE-CR to a 12-week center-based exercise cardiac rehabilitation program in people with cardiovascular disease [19]. At the 12-week follow-up, REMOTE-CR was shown to be noninferior to center-based exercise cardiac rehabilitation on maximum oxygen uptake (VO_2_max; adjusted mean difference [AMD] 0.51, 95% CI 0.97-1.98 mL/kg/minute). At longer-term follow-up (24 weeks), participants allocated to REMOTE-CR were also more likely to be participating in physical activity than those allocated to the center-based program (ie, less sedentary time: AMD –62, 95% CI –118 to –5 minutes/day) [19]. Importantly, per capita costs for delivering REMOTE-CR were 70% lower than center-based exercise cardiac rehabilitation, and although more adverse events were self-reported by the REMOTE-CR group during the intervention period, none were severe, and the majority (42/50, 84%) were not related to program participation. The same approach could be adapted for use in a cardio-oncology setting. However, the extent to which it would be feasible, safe, and effective (ie, positively impact fitness outcomes) for survivors of breast cancer at risk of cardiotoxicity, given their unique needs and risk profile, is unknown.

The overall aim of this study is to determine the feasibility of the REMOTE-COR-B program, a smartphone-based telerehabilitation exercise program for survivors of breast cancer at risk of cardiotoxicity. The secondary objectives of this trial are to determine satisfaction with and usability of REMOTE-COR-B, as well as potential effects on cardiovascular fitness that can be used to inform future, adequately powered trials.

## Methods

### Ethical Considerations

Ethics clearance was obtained by the Peter MacCallum Cancer Centre Ethics Committee (HREC/60412/PMCC-2020). Informed consent will be obtained from all participants involved in the trial. Participants will be assigned a study ID number. All study materials will be coded with the ID number only. Only the research team will have access to the study database, which contains the information needed to link ID numbers with identifiable information. Participants will be provided with an AUD $25 (US $16.32) gift card at the end of each assessment as an incentive to complete outcome assessments and in acknowledgement of their time. Participants will also be reimbursed for travel expenses or parking costs for attendance at each appointment.

### Trial Design

A single-arm feasibility study will be conducted to determine the feasibility, safety, and preliminary efficacy of REMOTE-COR-B (protocol version 7; date August 24, 2023). The trial has been prospectively registered on the Australian New Zealand Clinical Trials Registry (ACTRN12621001557820). The study protocol is reported in accordance with the SPIRIT (Standard Protocol Items: Recommendations for Interventional Trials) guidelines [20], and the intervention is described according to the Consensus on Exercise Reporting Template (CERT) [21]. Study materials are available on the Open Science Framework [22].

### Eligibility and Recruitment

The inclusion and exclusion criteria are provided in Textbox 1. Eligibility will be confirmed using medical data and patient interviews, as appropriate.

**The inclusion and exclusion criteria for the study.**

**Inclusion criteria**

- A diagnosis of stage I-III breast cancer.
- At risk of cardiotoxicity according to predetermined criteria (taking treatment type and dose as well as other common cardiovascular disease risk factors into account, including age, obesity, and the presence of other comorbidities; Figure S1 in Multimedia Appendix 1 [3,6,23-27]).
- Completion of primary definitive anticancer therapy within the last 24 months (which may be surgery, radiotherapy, or chemotherapy depending on the treatment pathway; participants who received both adjuvant and neoadjuvant treatment are eligible).
- Generally participating in less than the REMOTE-COR-B exercise target (ie, <150 minutes of moderate to vigorous intensity aerobic activity per week over <3 sessions per week) [13,14,28].
- An Eastern Cooperative Oncology Group (ECOG) performance status of 0-2 [28]
- Having sufficient reading and writing English skills is required for understanding the participant information sheet and study participation instructions.

**Exclusion criteria**

- Participants who have been diagnosed with metastatic (stage IV) or recurrent breast cancer.
- Those who have a medical condition where exercise or cardiopulmonary exercise testing is contraindicated (eg, unstable angina, uncontrolled heart failure, or asthma) [21].
- Having an implanted cardiac device [21].
- Being unable to provide informed consent.
- Being unable to fully participate in study assessments due to cognitive or physical impairment.
- Participating in another exercise study or exercise program with similar goals.
- Participating in a clinical trial that presents safety or contamination issues for either trial (to be assessed by the Steering Committee).

Eligible participants will be identified by the breast cancer clinical and research staff at the Peter MacCallum Cancer Centre and the Royal Melbourne Hospital. Potentially eligible patients will receive an email through the REDCap (Research Electronic Data Capture [29,30]) platform and will be provided with an information pack (including a study flyer and participant information and consent form). Patients can indicate their interest in the trial through the REDCap platform or by direct contact with the research team. Trial staff will contact interested patients to provide a verbal explanation of the project and its procedures, answer patients’ questions, and complete the eligibility screening. Following confirmation of eligibility, trial staff will obtain informed consent to participate in the study through REDCap, and the baseline assessment will be scheduled. Participants can withdraw from the study at any time without reason or consequence. In addition, the investigator may discontinue a patient from the study at any time if the investigator considers it necessary for any reason.

### Adapting the REMOTE-CR Intervention

For this study, REMOTE-COR-B was adapted from the original REMOTE-CR platform, which is described in detail elsewhere [31]. In brief, the base platform comprises a smartphone and wearable sensor (currently compatible with BioHarness 3, Zephyr Technology, and H10, Polar Electro), as well as a custom-built smartphone app and web application. The platform facilitates remotely supervised exercise prescriptions that are delivered, monitored, and coached in real-time by an exercise professional. The participant’s heart rate and geospatial data are displayed in the smartphone app for self-monitoring and streamed to a web server for review by an exercise professional (along with single-lead electrocardiogram [ECG] data, which are not visible to the participant in the smartphone app). The participant can also use the app to report “red flag” symptoms during exercise (chest pain, breathlessness, and dizziness), allowing the exercise professional to stop the session, make contact, and direct as needed. The exercise professional provides individualized audio coaching, feedback, and social support throughout the session, which is delivered to participants through earphones (text-to-audio feature) to optimize usability. At the end of an exercise session, participants are prompted to report their perceived exertion using the app. Outside of real-time interaction, participants can record exercise training for self-monitoring, receive behavior change education through direct messaging, review all recorded exercise performance data, and set or review goals to encourage behavior change.

### Adaptations of REMOTE-CR for Breast Cancer

**Figure 1:**
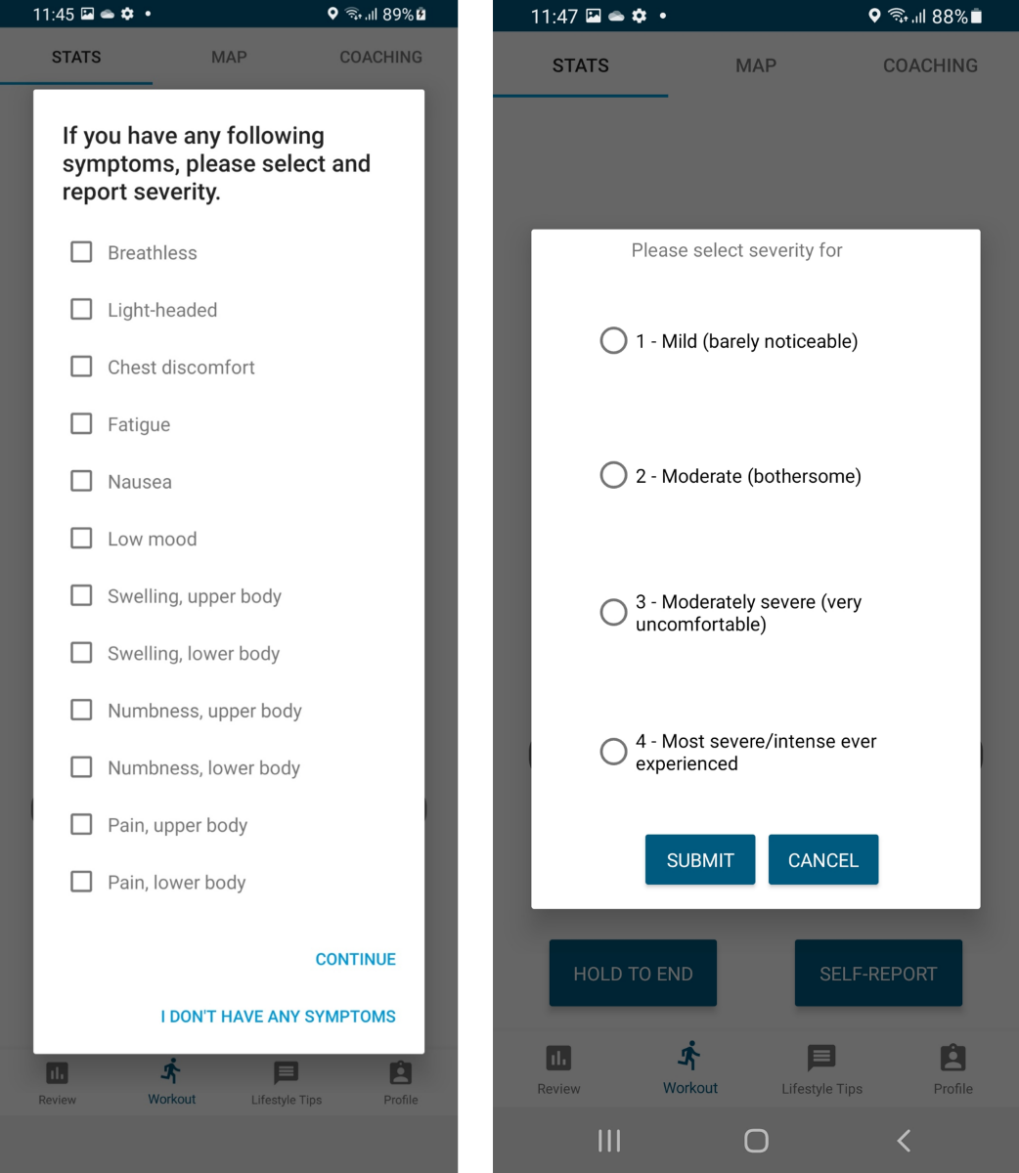
Expanded symptom reporting list in REMOTE-COR-B: a single-arm feasibility trial for patients with breast cancer.

**Figure 2:**
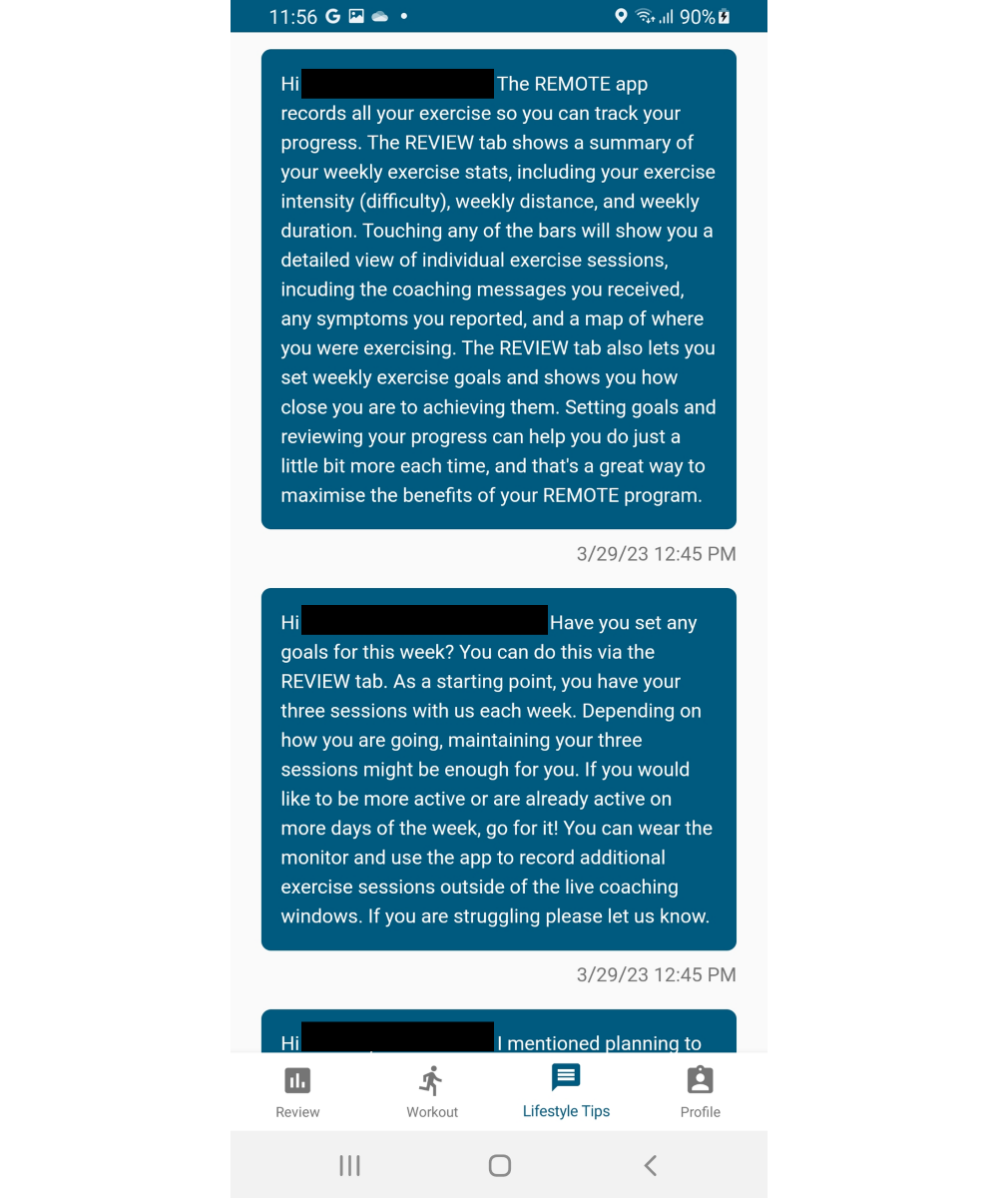
Example of behavior change support in REMOTE-COR-B: a single-arm feasibility trial for patients with breast cancer.

Following consultation with consumer representatives and the advisory committee, the following changes were made to the base platform for the REMOTE-COR-B trial: (1) expansion of the symptom reporting list to include common breast cancer treatment-related side effects and symptoms (Figure 1); (2) prompts for participants to report their symptoms at the start of each exercise session; (3) ability to report Rating of Perceived Exertion (RPE) both during and after exercise to enhance tailoring of prescription and coaching; (4) expansion of the heart rate data display to include beats per minute (BPM; the raw measurement unit) in addition to heart rate reserve, to allow use when a lack of maximal exercise testing precludes calculation of heart rate reserve; (5) revisions to the behavior change education to provide additional positive reinforcement, breast cancer–specific content, and messages focused on planning, habit formation, and autonomous motivation; and (6) alternative options for wearing the heart rate monitor if chest irritation or pain is an issue (ie, delay enrollment post treatment, use an adhesive to place the sensor on an unaffected area or at a different location that yields sufficient signal quality, or monitor intensity exclusively through RPE).

### The Remote-COR-B Intervention

The REMOTE-COR-B intervention will be delivered over a period of 8 weeks and involves remotely delivered individualized exercise prescriptions, real-time supervision, and behavior change support (eg, goal setting and supportive messages). The aim of the intervention is for participants to attend 3 remotely monitored aerobic exercise sessions per week, with self-directed exercise encouraged (where appropriate) on ≥2 other days to align with current aerobic recommendations for patients with cancer (ie, 150 minutes of moderate to vigorous intensity activity per week) [13,14,28]. The REMOTE-COR-B trial will emphasize aerobic activity, given the focus of the intervention on improving cardiovascular fitness [14,28]. The intervention is summarized below and described in detail according to the CERT requirements in Table S1 in Multimedia Appendix 2 [21,32,33].

### Exercise Prescription

Each participant will be provided with an individualized and progressive exercise prescription based on their age, previous and recent exercise habits, motivation, personal goals, attitudes, values, treatment, and support. Exercise prescription components will include frequency (3 remotely monitored sessions per week), duration (20-60 minutes per session), and intensity (moderate: 40%-60% heart rate reserve [HRR] and RPE 3-5 [34]), with the aim of increasing peak oxygen consumption (VO_2_peak). Intensity will be monitored through a combination of HRR and RPE if complete baseline cardiopulmonary exercise testing (CPET) is available (more details are provided in the “Secondary Outcomes” section), or BPM and RPE if baseline CPET is unavailable.

The progression of exercise prescription components will typically occur in the following order: duration, intensity, frequency (additional to remotely monitored sessions), and will be gradually increased as tolerated by the individual, with consideration to symptom status, fitness level, exercise response (based on heart rate data and RPE), and each participant’s goals [35]. During the first half of the intervention (ie, weeks 1-4), exercise intensity targets will typically range from RPE 3 to 4 (“moderate” to “somewhat hard”) and between 40% and 50% HRR [34]. In the second half of the intervention (ie, weeks 5-8), exercise intensity targets will typically range from RPE 4 to 5 (“somewhat hard” to “hard”) and between 50% and 60% HRR [34]. The preferred mode of exercise is walking, though participants may choose other land-based aerobic activities (eg, cycling, jogging, and exercise videos).

### Remote Monitoring and Supervision

Participants will be able to attend remotely monitored exercise sessions during set operating hours each week, scheduled in both the morning (eg, between 6 AM and 10 AM) and evening (eg, between 5:30 PM and 7:30 PM). Participants can complete exercise in any location with an active broadband connection (mobile, Wi-Fi, or Bluetooth). The REMOTE-COR-B mobile app collects heart rate and single-lead ECG through a Polar H10 heart rate transmitter (loaned to participants throughout the study), location, distance, and speed through location services integration, as well as symptoms and RPE through self-report. These data are then transmitted to a cloud-based server and displayed on a companion web application in real-time. Exercise professionals remotely monitor all data in real-time through the web application, provide participants with real-time individualized coaching feedback and support through their smartphone app (through alerts, messages, or telephone calls), respond to adverse events if required, provide postexercise feedback, and modify exercise prescriptions as needed.

### Behavior Change Support

Participants will receive behavior change education and support during the 8-week intervention period through push notifications (2-4 per week) delivered through the REMOTE-COR-B mobile app (Figure 2). The messages are based on social cognitive [36], self-determination [37], and habit theories [38] (Table S1 in Multimedia Appendix 2 outlines the specific strategies). Additionally, participants are encouraged to use the built-in data visualization features of the app to review their exercise performance data and set and review goals to encourage behavior change.

### Outcomes and Assessment Timing

Data relating to intervention delivery outcomes (eg, adherence, feasibility, and safety) will be collected during the 8-week intervention (Table 1). Satisfaction and usability will be assessed post intervention. Objective and patient-reported efficacy outcomes will be assessed at baseline (within 24 months post completion of primary definition treatment), post intervention (2-month postbaseline assessment), and at follow-up (5-month postbaseline assessment). Demographic and medical data will be collected at baseline through self-reports and medical records. Criteria of success for core feasibility, usability, safety, and efficacy outcomes have been prespecified and are outlined below.

**Table 1:** Schedule of enrollment, intervention, and assessments for REMOTE-COR-B: a single-arm feasibility trial for patients with breast cancer (based on the SPIRIT [Standard Protocol Items: Recommendations for Interventional Trials] guidelines) [20].

Time point		Study period					
		Enrollment (prebaseline)	Intervention period				Follow-up
			Baseline (0 months)	Intervention (2 months)	Postintervention (2-month postbaseline)	Follow-up (5-month postbaseline)	
**Enrollment**							
	Cardiotoxicity screen (including treatment information)	✓					
	Eligibility screen	✓					
	Informed consent	✓					
**Interventions**							
	Single-arm exercise			✓			
**Assessments**							
	Demographic information	✓	✓				
	Intervention adherence			✓			
	Feasibility	✓	✓		✓	✓	
	Safety		✓	✓	✓	✓	
	Intervention satisfaction				✓		
	Intervention usability				✓		
	CPET^a^		✓		✓	✓	
	ISWT^b^		✓		✓	✓	
	Quality of life		✓		✓	✓	
	Fatigue		✓		✓	✓	
	Self-reported exercise		✓		✓	✓	
	Psychological mechanisms		✓		✓	✓	

^a^CPET: cardiopulmonary exercise testing.

^b^ISWT: incremental shuttle walk test.

### Primary Outcome

Adherence will be assessed through the number of remotely monitored exercise sessions attended compared to the trial target (ie, 3 sessions per week). Previous research suggests ≥70% adherence is satisfactory to achieve fitness gains [8,12]. As such, participants will be considered adherent to the intervention if they complete ≥17 of 24 remotely monitored exercise sessions.

### Secondary Outcomes

#### Trial Feasibility

Trial feasibility is assessed based on rates of screening, consent, device ownership, retention, and missing data across all assessment points. Prespecified cut points indicating feasibility include cardiac dysfunction risk level is accessible for ≥80% of cases identified; ≥60% of those invited to participate agree to complete eligibility screening [39]; the recruitment goal is reached within the allotted time (1 year); retention is ≥60%; and outcome data are collected for ≥80% of enrolled participants at both the postintervention and follow-up assessments (this is in line with retention observed by the chief investigator Maddison et al [19] in the REMOTE-CR trial).

#### Safety

Adverse events will be assessed through the frequency and severity of adverse events reported during remotely monitored exercise sessions, any unsupervised exercise sessions, and exercise testing sessions (as outlined in the “Cardiovascular Fitness: VO_2_peak” section). Severity will be graded according to the Common Terminology Criteria for Adverse Events 5.0 and the National Health and Medical Research Council guidelines for safety monitoring and reporting [32,40]. Participants will be instructed to immediately report any adverse events that occur during the intervention period (during either supervised or unsupervised exercise sessions) to the study staff (ie, exercise professionals or study coordinators); these will be recorded. All adverse events reported to study staff will be communicated to the study coordinator and the coordinating principal investigator as soon as possible and recorded. The coordinating principal investigator will be responsible for ensuring serious adverse events are reported to the ethics committee and trial sponsor within the appropriate time frames. Participants can also retrospectively report adverse events during the postintervention and follow-up surveys. The trial will be deemed safe if no grade ≥3 adverse events attributed to participating in the research project are reported. During the intervention period, participants can also self-report symptoms (new, ongoing, or worsening) through the smartphone app at the beginning and end of every exercise session (supervised and unsupervised). Any changes in symptomology will also be recorded.

#### Satisfaction and Usability

Satisfaction with the intervention and perceived intervention usability will be assessed using the validated 8-item Client Satisfaction Questionnaire (0-32) [41] and the 10-item System Usability Scale (0-100) [42]. The intervention will be deemed satisfactory and usable if mean scores are ≥24 and ≥68, respectively. Issues with wearing the heart rate monitor due to cancer treatment-related discomfort will also be discussed and recorded by the study coordinator in a study-specific form.

#### Cardiovascular Fitness: VO2peak

Cardiovascular fitness (VO_2_peak) will be assessed through CPET and an incremental shuttle walk test (ISWT). The CPET will occur at the Peter MacCallum Cancer Centre (conducted by Peter MacCallum staff) and will involve participants exercising on an exercise bike against increasing resistance, during which time gas exchange analysis will be conducted to measure anaerobic threshold and VO_2_peak. Blood pressure and 12-lead ECG will be monitored for signs of an adverse cardiac response. VO_2_peak is a gold-standard measure for objectively assessing cardiorespiratory fitness and is an important clinical end point in this population because it is a strong predictor of adverse cardiovascular events and mortality [11]. A change in VO_2_peak as small as 6% can be clinically meaningful in terms of cardiovascular outcomes among patients with chronic systolic heart failure [43]. We expect an average improvement in VO_2_peak of ≥10%, as this is comparable to what has been achieved in posttreatment supervised exercise interventions among patients with breast cancer [44-46]. Additionally, this level of change is used clinically in a heart failure setting as indicating a clinically meaningful improvement in outcomes [47,48] and is associated with improved health-related quality of life in patients with breast cancer [46].

The ISWT is a valid and reliable field walking test to assess functional exercise capacity [49,50] and is shown to be moderately correlated with CPET-assessed VO_2_peak (*r*=0.61) [51]. Due to possible COVID-19–related difficulties with performing CPETs on all participants, an ISWT will also be performed (conducted by trial staff), as the equipment is easily transportable and the test can be completed outdoors (potentially necessary if a home visit is required due to COVID-19 restrictions and a suitable indoor location is not available). Participants are required to walk around 2 cones placed 9 meters apart (a total of 10 meters of flat course) in time to a set of auditory beeps. Initially, the walking speed is very slow, but each minute, the required walking speed progressively increases. The test concludes when the participant cannot achieve the required speed, experiences clinical indications for test termination, or wishes to stop. The number of shuttles is recorded. Only standardized instructions will be used [50], the walking track will be kept the same for all tests for a participant, and no encouragement will be given throughout. An improvement of ≥48 meters from baseline to postintervention will be considered clinically significant [49]. The intervention will be deemed potentially efficacious for improving cardiovascular fitness if clinically significant changes in VO_2_peak (≥10%) or meters walked in the ISWT are achieved.

#### Quality of Life

Quality of life will be assessed using the validated 38-item Functional Assessment of Cancer Therapy-Breast (FACT-B) questionnaire [52]. Total health-related quality of life (0-148) and subscale scores (physical, social, emotional, and functional well-being) will be calculated. A clinically important change in total quality of life has been defined as a 6% increase between baseline and postintervention [53].

#### Fatigue

Fatigue will be assessed using the validated 13-item Functional Assessment of Chronic Illness Therapy-Fatigue Scale (FACIT-Fatigue) questionnaire [54]. All items are summed to create a single fatigue score (0-52). A clinically important change in the FACIT-Fatigue has been defined as a 6% increase between baseline and postintervention [53,55].

#### Self-Reported Exercise

Self-reported exercise will be assessed using the validated 3-item Godin Leisure-Time Exercise Questionnaire (GLTEQ) [56]. Using the GLTEQ, participants self-report how often in the previous 7 days they completed ≥15 minutes of strenuous, moderate, and light physical activity. The number of bouts at each intensity is then multiplied by the corresponding MET value and summed to create a Leisure Score Index (LSI). The proportion of participants considered sufficiently active will also be reported, determined based on prespecified LSI scores (<14 units=insufficiently active or sedentary; between 14 and 23 units=moderately active; and ≥24 units=active or “meeting criteria for being physically active”) [56,57].

#### Psychological mechanisms

Potential mediators of intervention effects will be assessed, including self-efficacy (Multidimensional Self-Efficacy for Exercise Scale [58]), habit strength (self-reported habit index [59]), motivation type (Behavioural Regulation Questionnaire [60]), and autonomy-supportive exercise environment (The Perceived Environmental Supportiveness Scale [61]).

### Data Analysis

#### Sample Size

We aim to recruit 40 participants over the course of 12 months. This sample size is considered sufficient to test the feasibility of delivering the intervention and other key trial parameters [62]. Notably, with this sample size, we will also have reasonable precision for estimating intervention adherence. For example, with a sample size of 40, if the average adherence rate observed in the study is 70%, we can be sure with 95% confidence that the true population proportion is between 56% and 85%; the 95% CI is ≥14.2. Given the potential reach of the intervention and the fact that adherence to current technology-based behavior change programs is 50% [39], this finding would warrant further investigation. The target sample size is also equivalent to approximately 20%-30% of the sample size needed to adequately power (80%) a future definitive trial to demonstrate noninferiority in VO_2_peak changes between groups (a *P* value of .05); based on differences observed in our formative work [19] and a systematic review [8].

#### Statistical Methods

The data will be presented descriptively where appropriate; this includes most feasibility outcomes (eg, recruitment rate, proportion of missing data, usability, and satisfaction), including the primary outcome (ie, adherence to the intervention), and safety outcomes (eg, number of adverse events, proportion of serious adverse events).

Changes in VO_2_peak, patient-reported outcomes (quality of life and fatigue), and physical activity behavior will be examined in exploratory analyses at all assessment time points using mixed model repeated measures analyses. Unadjusted and age- and treatment pathway–adjusted models will be conducted with standardized coefficients reported with 95% CIs to aid interpretation of clinical significance rather than *P* values given a lack of statistical power. The choice of modeling link will be informed by residual diagnostics. The data for withdrawn participants collected before study withdrawal will be retained for use in intention-to-treat analyses, unless requested otherwise by the participant.

Further exploratory analyses may include (1) examining changes in the proposed psychological mechanisms (eg, self-efficacy) from baseline to postintervention; (2) examining associations between the proposed mechanisms and the study outcomes (eg, adherence to exercise sessions and cardiorespiratory fitness); and (3) examining associations between participant characteristics (potential moderators) and intervention adherence (sessions and intensity), as well as changes in VO_2_peak. Given the small sample size and absence of a control group, a formal mediation analysis or moderation analysis will not be undertaken (as per Baron and Kenny [63]). However, the collection of these data will assist with determining the feasibility of mediator and moderator assessment in a larger trial, where a formal mediation analysis and moderator analysis may be undertaken.

### Data Management

The data will be preferentially recorded in electronic case record forms (CRF) and through surveys using REDCap data management software, only accessible to trial staff. Hard copy forms will also be available if necessary (stored in a locked filing cabinet). Data collected through hard copy will be scanned and uploaded to REDCap and the secure study network drive as soon as possible. Hard copies will be shredded once the data are safely stored electronically (including a scan of the original). All data will be exported from REDCap at the end of the trial and stored on a password-protected network drive at the University of Melbourne for at least 5 years after the publication of the results. Deidentified data may be shared indefinitely through sites like Open Science or Figshare to ensure scientific transparency and to share knowledge with others.

## Results

The recruitment for this trial began in March 2023, and 7 participants had been recruited as of the submission of the manuscript. Recruitment issues were encountered early, and changes to the prespecified protocol (ACTRN12621001557820) have been made to aid recruitment. The original eligibility criteria for the trial required participants to have completed primary, definitive anticancer therapy within 3-12 weeks. In addition, only anthracyclines and no other types of chemotherapy were considered in the cardiotoxic risk assessment. After screening potential participants, it became clear that recruitment would be slower than expected and that many patients at risk of poor heart health would not be eligible for the trial. Before enrolling the first participant, the eligibility criteria were amended so that participants could be up to 6 months post definitive treatment, and a wider range of evidence-based risk factors for cardiotoxicity and cardiovascular disease would be considered (Figure S1 in Multimedia Appendix 1 [3,6,23-27]). A second objective fitness test (the ISWT) was also added, which can be conducted outside of the hospital setting in case COVID-19 disruptions impact access to CPET assessments. Following these changes, the first participant was enrolled in March 2023.

Additional changes have since been made to the protocol to further aid recruitment. These include expanding screening opportunities to identify potentially eligible participants, streamlining the recruitment procedure through REDCap to reduce burden on hospital staff, and expanding eligibility criteria to include patients up to 24 months post definitive treatment. This amendment was recently approved, and the estimated completion date for the project is October 2024, with results expected to be published in mid-2025. Any future protocol amendments will be reported in the main outcomes publication and registered on the Australian New Zealand Clinical Trials Registry.

## Discussion

The original intervention underpinning this trial has demonstrated efficacy and noninferiority compared to gold-standard in-clinic cardiac rehabilitation [19]. The adaptation of this intervention for patients with breast cancer who are at risk of cardiotoxicity is a novel and promising approach to providing accessible exercise therapy. This trial will address the extent to which this approach is satisfactory to patients with breast cancer, safe, and potentially effective, given the unique needs and risk profile of this group. The findings will be used to inform features of a future, adequately powered efficacy trial, including but not limited to eligibility and recruitment strategy, app features and functions, and delivery preferences (eg, session timing) [64]. Trial findings might also be used to inform the implementation of this approach in breast cancer services, given that remote strategies are needed now and the strong evidence-based basis behind the base software (REMOTE-CR). By publishing this protocol, we aim to ensure transparency around prespecified outcome criteria, inform interested parties of the upcoming trial, and aid replication and critical review of study methodology.

## Appendix

Multimedia Appendix 1 Cardiotoxicity criteria.

Multimedia Appendix 2 Exercise prescription principles of the REMOTE-COR-B trial (based on Consensus on Exercise Reporting Template).

## Abbreviations

AMD: adjusted mean difference
BPM: beats per minute
CERT: Consensus on Exercise Reporting Template
CPET: cardiopulmonary exercise testing
CRF: case record forms
ECG: electrocardiogram
FACIT-Fatigue: Functional Assessment of Chronic Illness Therapy-Fatigue Scale
FACT-B: Functional Assessment of Cancer Therapy-Breast
GLTEQ: Godin Leisure-Time Exercise Questionnaire
HER: human epidermal growth factor receptor
HRR: heart rate reserve
ISWT: incremental shuttle walk test
LSI: Leisure Score Index
MET-hrs: metabolic equivalent task hours
REDCap: Research Electronic Data Capture
RPE: Rating of Perceived Exertion
SPIRIT: Standard Protocol Items: Recommendations for Interventional Trials
VO2max: maximum oxygen uptake
VO2peak: peak oxygen consumption

## Data Availability

The data sets generated or analyzed during this study will be available from the corresponding author upon reasonable request.

## References

[1] Australian Institute of Health and Welfare (2012). Breast Cancer in Australia: An Overview.

[2] Lovelace DL, McDaniel LR, Golden D (2019). Long-term effects of breast cancer surgery, treatment, and survivor care. J Midwifery Womens Health.

[3] Armenian SH, Lacchetti C, Barac A, Carver J, Constine LS, Denduluri N, Dent S, Douglas PS, Durand J, Ewer M, Fabian C, Hudson M, Jessup M, Jones LW, Ky B, Mayer EL, Moslehi J, Oeffinger K, Ray K, Ruddy K, Lenihan D (2017). Prevention and monitoring of cardiac dysfunction in survivors of adult cancers: American Society of Clinical Oncology Clinical Practice Guideline. J Clin Oncol.

[4] (2021). Cardiovascular diseases (CVDs). World Health Organisation.

[5] Bradshaw PT, Stevens J, Khankari N, Teitelbaum SL, Neugut AI, Gammon MD (2016). Cardiovascular disease mortality among breast cancer survivors. Epidemiology.

[6] Koene RJ, Prizment AE, Blaes A, Konety SH (2016). Shared risk factors in cardiovascular disease and cancer. Circulation.

[7] Smith LA, Cornelius VR, Plummer CJ, Levitt G, Verrill M, Canney P, Jones A (2010). Cardiotoxicity of anthracycline agents for the treatment of cancer: systematic review and meta-analysis of randomised controlled trials. BMC Cancer.

[8] Scott JM, Nilsen TS, Gupta D, Jones LW (2018). Exercise therapy and cardiovascular toxicity in cancer. Circulation.

[9] Ross R, Blair SN, Arena R, Church TS, Després JP, Franklin BA, Haskell WL, Kaminsky LA, Levine BD, Lavie CJ, Myers J, Niebauer J, Sallis R, Sawada SS, Sui X, Wisløff U (2016). Importance of assessing cardiorespiratory fitness in clinical practice: a case for fitness as a clinical vital sign: a scientific statement from the American Heart Association. Circulation.

[10] Early Breast Cancer Trialists' Collaborative Group (EBCTCG) (2005). Effects of chemotherapy and hormonal therapy for early breast cancer on recurrence and 15-year survival: an overview of the randomised trials. Lancet.

[11] Palomo A, Ray RM, Johnson L, Paskett E, Caan B, Jones L, Okwuosa T (2017). Associations between exercise prior to and around the time of cancer diagnosis and subsequent cardiovascular events in women with breast cancer: a Women's Health Initiative (WHI) analysis. J Am Coll Cardiol.

[12] Buffart LM, Kalter J, Sweegers MG, Courneya KS, Newton RU, Aaronson NK, Jacobsen PB, May AM, Galvão Daniel A, Chinapaw MJ, Steindorf K, Irwin ML, Stuiver MM, Hayes S, Griffith KA, Lucia A, Mesters I, van Weert E, Knoop H, Goedendorp MM, Mutrie N, Daley AJ, McConnachie A, Bohus M, Thorsen L, Schulz K, Short CE, James EL, Plotnikoff RC, Arbane G, Schmidt ME, Potthoff K, van Beurden M, Oldenburg HS, Sonke GS, van Harten WH, Garrod R, Schmitz KH, Winters-Stone KM, Velthuis MJ, Taaffe DR, van Mechelen W, Kersten M, Nollet F, Wenzel J, Wiskemann J, Verdonck-de Leeuw IM, Brug J (2017). Effects and moderators of exercise on quality of life and physical function in patients with cancer: An individual patient data meta-analysis of 34 RCTs. Cancer Treat Rev.

[13] Cormie P, Atkinson M, Bucci L, Cust A, Eakin E, Hayes S, McCarthy S, Murnane A, Patchell S, Adams D (2018). Clinical oncology society of Australia position statement on exercise in cancer care. Med J Aust.

[14] Hayes SC, Newton RU, Spence RR, Galvão DA (2019). The exercise and sports science Australia position statement: exercise medicine in cancer management. J Sci Med Sport.

[15] Hardcastle SJ, Cohen PA (2017). Effective physical activity promotion to survivors of cancer is likely to be home based and to require oncologist participation. J Clin Oncol.

[16] Short CE, James EL, Stacey F, Plotnikoff RC (2013). A qualitative synthesis of trials promoting physical activity behaviour change among post-treatment breast cancer survivors. J Cancer Surviv.

[17] Beatty AL, Fukuoka Y, Whooley MA (2013). Using mobile technology for cardiac rehabilitation: a review and framework for development and evaluation. J Am Heart Assoc.

[18] Maddison R, Pfaeffli L, Whittaker R, Stewart R, Kerr A, Jiang Y, Kira G, Leung W, Dalleck L, Carter K, Rawstorn J (2015). A mobile phone intervention increases physical activity in people with cardiovascular disease: results from the HEART randomized controlled trial. Eur J Prev Cardiol.

[19] Maddison R, Rawstorn JC, Stewart RAH, Benatar J, Whittaker R, Rolleston A, Jiang Y, Gao L, Moodie M, Warren I, Meads A, Gant N (2019). Effects and costs of real-time cardiac telerehabilitation: randomised controlled non-inferiority trial. Heart.

[20] Chan AW, Tetzlaff JM, Altman DG, Laupacis A, Gøtzsche PC, Krleža-Jerić K, Hróbjartsson A, Mann H, Dickersin K, Berlin JA, Doré CJ, Parulekar WR, Summerskill WSM, Groves T, Schulz KF, Sox HC, Rockhold FW, Rennie D, Moher D (2013). SPIRIT 2013 statement: defining standard protocol items for clinical trials. Ann Intern Med.

[21] Slade SC, Dionne CE, Underwood M, Buchbinder R (2016). Consensus on Exercise Reporting Template (CERT): explanation and elaboration statement. Br J Sports Med.

[22] Short, CE, Jones, TL (2024). REMOTE-COR-B.

[23] Lyon Alexander R, Dent Susan, Stanway Susannah, Earl Helena, Brezden-Masley Christine, Cohen-Solal Alain, Tocchetti Carlo G, Moslehi Javid J, Groarke John D, Bergler-Klein Jutta, Khoo Vincent, Tan Li Ling, Anker Markus S, von Haehling Stephan, Maack Christoph, Pudil Radek, Barac Ana, Thavendiranathan Paaladinesh, Ky Bonnie, Neilan Tomas G, Belenkov Yury, Rosen Stuart D, Iakobishvili Zaza, Sverdlov Aaron L, Hajjar Ludhmila A, Macedo Ariane V S, Manisty Charlotte, Ciardiello Fortunato, Farmakis Dimitrios, de Boer Rudolf A, Skouri Hadi, Suter Thomas M, Cardinale Daniela, Witteles Ronald M, Fradley Michael G, Herrmann Joerg, Cornell Robert F, Wechelaker Ashutosh, Mauro Michael J, Milojkovic Dragana, de Lavallade Hugues, Ruschitzka Frank, Coats Andrew J S, Seferovic Petar M, Chioncel Ovidiu, Thum Thomas, Bauersachs Johann, Andres M Sol, Wright David J, López-Fernández Teresa, Plummer Chris, Lenihan Daniel (2020). Baseline cardiovascular risk assessment in cancer patients scheduled to receive cardiotoxic cancer therapies: a position statement and new risk assessment tools from the Cardio-Oncology Study Group of the Heart Failure Association of the European Society of Cardiology in collaboration with the International Cardio-Oncology Society. Eur J Heart Fail.

[24] Lyon Alexander R, López-Fernández Teresa, Couch Liam S, Asteggiano Riccardo, Aznar Marianne C, Bergler-Klein Jutta, Boriani Giuseppe, Cardinale Daniela, Cordoba Raul, Cosyns Bernard, Cutter David J, de Azambuja Evandro, de Boer Rudolf A, Dent Susan F, Farmakis Dimitrios, Gevaert Sofie A, Gorog Diana A, Herrmann Joerg, Lenihan Daniel, Moslehi Javid, Moura Brenda, Salinger Sonja S, Stephens Richard, Suter Thomas M, Szmit Sebastian, Tamargo Juan, Thavendiranathan Paaladinesh, Tocchetti Carlo G, van der Meer Peter, van der Pal Helena J H (2022). 2022 ESC Guidelines on cardio-oncology developed in collaboration with the European Hematology Association (EHA), the European Society for Therapeutic Radiology and Oncology (ESTRO) and the International Cardio-Oncology Society (IC-OS). Eur Heart J.

[25] Zamorano Jose Luis, Lancellotti Patrizio, Rodriguez Muñoz Daniel, Aboyans Victor, Asteggiano Riccardo, Galderisi Maurizio, Habib Gilbert, Lenihan Daniel J, Lip Gregory Y H, Lyon Alexander R, Lopez Fernandez Teresa, Mohty Dania, Piepoli Massimo F, Tamargo Juan, Torbicki Adam, Suter Thomas M (2016). 2016 ESC Position Paper on cancer treatments and cardiovascular toxicity developed under the auspices of the ESC Committee for Practice Guidelines:  The Task Force for cancer treatments and cardiovascular toxicity of the European Society of Cardiology (ESC). Eur Heart J.

[26] Bovelli D, Plataniotis G, Roila F, ESMO Guidelines Working Group (2010). Cardiotoxicity of chemotherapeutic agents and radiotherapy-related heart disease: ESMO Clinical Practice Guidelines. Ann Oncol.

[27] Florescu Maria, Cinteza Mircea, Vinereanu Dragos (2013). Chemotherapy-induced cardiotoxicity. Maedica (Bucur).

[28] Campbell KL, Winters-Stone KM, Wiskemann J, May AM, Schwartz AL, Courneya KS, Zucker DS, Matthews CE, Ligibel JA, Gerber LH, Morris GS, Patel AV, Hue TF, Perna FM, Schmitz KH (2019). Exercise guidelines for cancer survivors: consensus statement from international multidisciplinary roundtable. Med Sci Sports Exerc.

[29] Harris PA, Taylor R, Minor BL, Elliott V, Fernandez M, O'Neal L, McLeod L, Delacqua G, Delacqua F, Kirby J, Duda SN (2019). The REDCap consortium: building an international community of software platform partners. J Biomed Inform.

[30] Harris PA, Taylor R, Thielke R, Payne J, Gonzalez N, Conde JG (2009). Research Electronic Data Capture (REDCap)—a metadata-driven methodology and workflow process for providing translational research informatics support. J Biomed Inform.

[31] Rawstorn JC, Gant N, Meads A, Warren I, Maddison R (2016). Remotely delivered exercise-based cardiac rehabilitation: design and content development of a novel mHealth platform. JMIR Mhealth Uhealth.

[32] (2017). Common Terminology Criteria for Adverse Events (CTCAE) Version 5.0. US Department of Health and Human Services.

[33] Slade Susan C, Dionne Clermont E, Underwood Martin, Buchbinder Rachelle, Beck Belinda, Bennell Kim, Brosseau Lucie, Costa Leonardo, Cramp Fiona, Cup Edith, Feehan Lynne, Ferreira Manuela, Forbes Scott, Glasziou Paul, Habets Bas, Harris Susan, Hay-Smith Jean, Hillier Susan, Hinman Rana, Holland Ann, Hondras Maria, Kelly George, Kent Peter, Lauret Gert-Jan, Long Audrey, Maher Chris, Morso Lars, Osteras Nina, Peterson Tom, Quinlivan Ros, Rees Karen, Regnaux Jean-Philippe, Rietberg Marc, Saunders Dave, Skoetz Nicole, Sogaard Karen, Takken Tim, van Tulder Maurits, Voet Nicoline, Ward Lesley, White Claire (2016). Consensus on Exercise Reporting Template (CERT): modified Delphi study. Phys Ther.

[34] Norton K, Norton L, Sadgrove D (2010). Position statement on physical activity and exercise intensity terminology. J Sci Med Sport.

[35] American College of Sports Medicine (2018). ACSM's Guidelines for Exercise Testing and Prescription, 10th Edition.

[36] Bandura A (2004). Health promotion by social cognitive means. Health Educ Behav.

[37] Deci EL, Ryan RM (2008). Self-determination theory: a macrotheory of human motivation, development, and health. Can Psychol / Psychologie canadienne.

[38] Gardner B, Lally P, Wardle J (2012). Making health habitual: the psychology of 'habit-formation' and general practice. Br J Gen Pract.

[39] Kelders SM, Kok RN, Ossebaard HC, Van Gemert-Pijnen JEWC (2012). Persuasive system design does matter: a systematic review of adherence to web-based interventions. J Med Internet Res.

[40] National Health and Medical Research Council (2016). Guidance: Safety Monitoring and Reporting in Clinical Trials Involving Therapeutic Goods.

[41] Larsen DL, Attkisson CC, Hargreaves WA, Nguyen TD (1979). Assessment of client/patient satisfaction: development of a general scale. Eval Program Plann.

[42] Brooke J, Jordan PW, McClelland IL, Thomas B, Weerdmeester BA (1996). SUS: a 'quick and dirty' usability scale. Usability Evaluation in Industry.

[43] Swank AM, Horton J, Fleg JL, Fonarow GC, Keteyian S, Goldberg L, Wolfel G, Handberg EM, Bensimhon D, Illiou MC, Vest M, Ewald G, Blackburn G, Leifer E, Cooper L, Kraus WE (2012). Modest increase in peak VO2 is related to better clinical outcomes in chronic heart failure patients: results from heart failure and a controlled trial to investigate outcomes of exercise training. Circ Heart Fail.

[44] Hornsby WE, Douglas PS, West MJ, Kenjale AA, Lane AR, Schwitzer ER, Ray KA, Herndon JE, Coan A, Gutierrez A, Hornsby KP, Hamilton E, Wilke LG, Kimmick GG, Peppercorn JM, Jones LW (2014). Safety and efficacy of aerobic training in operable breast cancer patients receiving neoadjuvant chemotherapy: a phase II randomized trial. Acta Oncol.

[45] Casla S, López-Tarruella S, Jerez Y, Marquez-Rodas I, Galvão DA, Newton RU, Cubedo R, Calvo I, Sampedro J, Barakat R, Martín M (2015). Supervised physical exercise improves VO2max, quality of life, and health in early stage breast cancer patients: a randomized controlled trial. Breast Cancer Res Treat.

[46] Courneya KS, Mackey JR, Bell GJ, Jones LW, Field CJ, Fairey AS (2003). Randomized controlled trial of exercise training in postmenopausal breast cancer survivors: cardiopulmonary and quality of life outcomes. J Clin Oncol.

[47] Edelmann F, Bobenko A, Gelbrich G, Hasenfuss G, Herrmann-Lingen C, Duvinage A, Schwarz S, Mende M, Prettin C, Trippel T, Lindhorst R, Morris D, Pieske-Kraigher E, Nolte K, Düngen HD, Wachter R, Halle M, Pieske B (2017). Exercise training in Diastolic Heart Failure (Ex-DHF): rationale and design of a multicentre, prospective, randomized, controlled, parallel group trial. Eur J Heart Fail.

[48] Kitzman DW, Brubaker P, Morgan T, Haykowsky M, Hundley G, Kraus WE, Eggebeen J, Nicklas BJ (2016). Effect of caloric restriction or aerobic exercise training on peak oxygen consumption and quality of life in obese older patients with heart failure with preserved ejection fraction: a randomized clinical trial. JAMA.

[49] Parreira VF, Janaudis-Ferreira T, Evans RA, Mathur S, Goldstein RS, Brooks D (2014). Measurement properties of the incremental shuttle walk test. A systematic review. Chest.

[50] Singh SJ, Morgan MD, Scott S, Walters D, Hardman AE (1992). Development of a shuttle walking test of disability in patients with chronic airways obstruction. Thorax.

[51] Granger CL, Denehy L, Parry SM, Martin J, Dimitriadis T, Sorohan M, Irving L (2015). Which field walking test should be used to assess functional exercise capacity in lung cancer? An observational study. BMC Pulm Med.

[52] Brady MJ, Cella DF, Mo F, Bonomi AE, Tulsky DS, Lloyd SR, Deasy S, Cobleigh M, Shiomoto G (1997). Reliability and validity of the Functional Assessment of Cancer Therapy-Breast quality-of-life instrument. J Clin Oncol.

[53] Eton DT, Cella D, Yost KJ, Yount SE, Peterman AH, Neuberg DS, Sledge GW, Wood WC (2004). A combination of distribution- and anchor-based approaches determined minimally important differences (MIDs) for four endpoints in a breast cancer scale. J Clin Epidemiol.

[54] Yellen SB, Cella DF, Webster K, Blendowski C, Kaplan E (1997). Measuring fatigue and other anemia-related symptoms with the Functional Assessment of Cancer Therapy (FACT) measurement system. J Pain Symptom Manage.

[55] Cella D, Eton DT, Lai JS, Peterman AH, Merkel DE (2002). Combining anchor and distribution-based methods to derive minimal clinically important differences on the Functional Assessment of Cancer Therapy (FACT) anemia and fatigue scales. J Pain Symptom Manage.

[56] Godin G (2011). The Godin-Shephard Leisure-Time Physical Activity Questionnaire. Health Fitness J Canada.

[57] Amireault S, Godin G, Lacombe J, Sabiston CM (2015). The use of the Godin-Shephard Leisure-Time Physical Activity Questionnaire in oncology research: a systematic review. BMC Med Res Methodol.

[58] Rodgers WM, Wilson PM, Hall CR, Fraser SN, Murray TC (2008). Evidence for a multidimensional self-efficacy for exercise scale. Res Q Exerc Sport.

[59] Gardner B, Abraham C, Lally P, de Bruijn GJ (2012). Towards parsimony in habit measurement: testing the convergent and predictive validity of an automaticity subscale of the Self-Report Habit Index. Int J Behav Nutr Phys Act.

[60] Wilson PM, Rodgers WM, Fraser SN (2002). Examining the psychometric properties of the Behavioral Regulation in Exercise Questionnaire. Meas Phys Educ Exerc Sci.

[61] Markland D, Tobin VJ (2010). Need support and behavioural regulations for exercise among exercise referral scheme clients: the mediating role of psychological need satisfaction. Psychol Sport Exerc.

[62] Thabane L, Ma J, Chu R, Cheng J, Ismaila A, Rios LP, Robson R, Thabane M, Giangregorio L, Goldsmith CH (2010). A tutorial on pilot studies: the what, why and how. BMC Med Res Methodol.

[63] Baron RM, Kenny DA (1986). The moderator-mediator variable distinction in social psychological research: conceptual, strategic, and statistical considerations. J Pers Soc Psychol.

[64] Blatch-Jones AJ, Pek W, Kirkpatrick E, Ashton-Key M (2018). Role of feasibility and pilot studies in randomised controlled trials: a cross-sectional study. BMJ Open.

