# Supplementary material for: Evaluating a Remotely Delivered Cardio-Oncology Rehabilitation Intervention for Patients With Breast Cancer (REMOTE-COR-B): Protocol for a Single-Arm Feasibility Trial
Source: JMIR Res Protoc. 2024 Apr 5;13:e53301. doi: 10.2196/53301 (PMC11031702; doi:10.2196/53301)
Supplement: Multimedia Appendix 1 [file resprot_v13i1e53301_app1.docx]

## Supplementary Figure 1. Cardiotoxicity Criteria

| **2 of 3 treatment types** | |
| --- | --- |
|  | Chemotherapy  *Including anthracyclines (e.g., doxorubicin, epirubicin); taxanes (e.g., paclitaxel, docetaxel); alkylating agents (e.g., cyclophosphamide).* |
|  | Radiation therapy where the heart is in the treatment field.  *(dose >30–35 Gy, dose per fraction >2 Gy).* |
|  | Human epidermal growth factor receptor 2 (HER2) targeted therapies  *(e.g., trastuzumab, pertuzumab).* |
| **OR** |  |
|  | 1 of the above treatment types |
| **AND** |  |
|  | Presence of any of the following risk factors: |
|  | - History of cardiovascular disease |
|  | - Older age at cancer treatment (≥ 60 years; ≥50 years for HER 2 therapies) |
|  | - Two or more cardiovascular risk factors, including:   - Smoking (current or quit < 10 years ago)   - High alcohol intake (> 14 standard drinks per week)   - Physically inactive   - Overweight (BMI ≥25) or Obese (BMI ≥30)   - Hypertension   - Diabetes   - Dyslipidaemia   - Chronic Kidney Disease   - Family history of premature cardiovascular disease (<50 years)   - Other  **_________** *provide clinical rationale* |

**Rationale for criteria:**

The above criteria were developed and refined based on review of the American Society of Clinical Oncology [3], European Society of Cardiology [23-25], and European Society for Medical Oncology [26] guidelines and consultation with clinical investigators. Individuals meeting these criteria are considered at increased risk of cardiotoxicity based on the treatments received, and/or the treatment received in combination with other risk factors.

Of note, our criteria are generally broader than the guidelines reviewed, which focus on defining high risk. For example, guidelines typically specify treatment with anthracycline-based chemotherapy, whereas we specify any chemotherapy agent (when used in combination with other known cardiotoxic treatments or in the presence of other cardiovascular risk factors). This approach was considered appropriate given evidence that other chemotherapy agents can be cardiotoxic [27] (albeit likely to a lesser extent), the well-established evidence regarding lifestyle risk factors and cardiovascular disease risk [6], the typical treatment practices and patient characteristics at our hospital sites, and the pilot nature of the trial. Detailed treatment information will be extracted from medical records and reported in upcoming manuscripts.
