# Supplementary material for: Evaluating a Remotely Delivered Cardio-Oncology Rehabilitation Intervention for Patients With Breast Cancer (REMOTE-COR-B): Protocol for a Single-Arm Feasibility Trial
Source: JMIR Res Protoc. 2024 Apr 5;13:e53301. doi: 10.2196/53301 (PMC11031702; doi:10.2196/53301)
Supplement: Multimedia Appendix 2 [file resprot_v13i1e53301_app2.docx]

## Supplementary Table 1. Exercise Prescription Principles of the REMOTE-COR-B Trial (based on Consensus on Exercise Reporting Template [21, 33])

| **Principles** | |
| --- | --- |
| **Application in REMOTE-COR-B** | **Notes** |
| **WHAT: materials** | |
| **Type of exercise equipment (Item 1)** | |
| Participants will be provided with the mobile phone app and a Polar Heart Rate Monitor (required for real time monitoring of exercise sessions).  Participants who do not have a smartphone or access to mobile data will be provided with a Samsung Galaxy A22-5G for use during the intervention. | No aerobic exercise equipment will be provided, the participants choice of aerobic mode may be dependent on the exercise equipment available to them.  Participants will also be encouraged to wear earphones during exercise sessions to optimise usability of the app. |
| **WHO: provider** | |
| **Qualifications of exercise instructor (Item 2)** | |
| All exercise sessions will be delivered by tertiary trained exercise professionals (an accredited exercise physiologist or physiotherapist) with experience and/or training in exercise oncology. | All exercise professionals will undergo study-specific training and will be provided with an intervention manual. |
| **HOW: delivery** | |
| **Group / individual (Item 3)** | |
| Intervention sessions (monitoring, real time feedback, and exercise prescription) will be delivered to participants on an individual basis.  Due the nature of the intervention which involves set hours of operation, multiple participants may attend live sessions and will be monitored simultaneously by an exercise professional. | Participants have the option to complete exercise sessions with family/friends if they would like to.  The number of participants attending exercise sessions simultaneously will be monitored to ensure there is an appropriate ratio of participants:exercise professionals and for future intervention planning purposes. |
| **Supervision (Item 4)** | |
| Through the REMOTE-COR-B platform, exercise prescription will be remotely delivered and supervised in real time by an exercise professional during set operating hours.  Set operating hours will be scheduled in the morning (e.g., Mon/Wed/Fri: 6:00am–10:00am) and evening (e.g., Tues/Thurs: 5:30pm–7:30pm). | Outside of set operating hours, participants can record exercise sessions using the REMOTE-COR-B platform. Real time supervision and coaching will not be available outside of scheduled hours.  Operating hours will be iteratively evaluated throughout the intervention to ensure efficient use of staffing resources. |
| **Adherence recording (Item 5)** | |
| Adherence to remotely monitored exercise sessions will be calculated based on the number of sessions completed (defined as the number of sessions attended during set operating hours) divided by the number of sessions prescribed (i.e., three per week).  Adherence data will be recorded via the REMOTE-COR-B platform and will be reported as a percentage. Data relating to exercise sessions (duration, intensity) will also be recorded via the platform. | Additional data on the number of sessions completed per participant may be available if participants choose to record additional sessions (outside of set operating hours) using the REMOTE-COR-B platform. Overall adherence to the exercises prescribed will be calculated using this data (i.e., number of sessions recorded divide by number prescribed). |
| **Motivational strategies and non-exercise components (Item 6)** | |
| Outside of real-time interaction, participants will receive behaviour change support through direct messaging based on social cognitive, self-determination, and habit theory.  Participants can (and are encouraged to) review all recorded exercise performance data and set/review goals to encourage behaviour change (see Figure 2). | The messages are designed to target the following constructs: outcome expectations, goals, relatedness, confidence, autonomy, habits, social support, identified and intrinsic motivation. Strategies used within messages include prompting goal setting, planning, social support, and self-monitoring, as well as setting graded tasks, encouraging the adoption of preparatory behaviours and exercise cues, and providing information on the consequences of the behaviour (including planning, goal setting and self-monitoring behaviours). |
| **Exercise Progression Decision Rules (Item 7a)** | |
| Exercise prescription will be progressed based on the principle of gradual progression. Progression may be achieved by increasing any FITT component.  For inactive individuals, the ACSM recommends initiating exercise at a light to moderate intensity and then increasing duration as tolerated by the individual. An increase of 5–10 minutes every 1–2 weeks in the initial phases of an exercise program is recommended. | Tolerance of exercise prescription will be evaluated based on individual characteristics including health status (e.g., symptoms and side-effects), fitness level, exercise response (based on objectively measured heart rate and self-reported RPE), and participant goals. |
| **Exercise Progression (Item 7b)** | |
| Progression of exercise prescription components will typically occur in the following order: duration, intensity, frequency (additional to remotely monitored sessions).  Progression from baseline exercise levels will be achieved by gradually increasing the duration of aerobic sessions, with the goal of progressing participants towards recommended levels of exercise (i.e., 150 minutes of moderate intensity exercise per week) through at least 3 remotely monitored sessions. Exercise intensity will also be gradually progressed throughout the intervention, and individual exercise intensity will be adapted during each session.  Weeks 1–4:  Duration will typically range from 20–45 minutes with an exercise intensity target from RPE 3–4 (“moderate” to “somewhat hard”) and/or 40%–50% HRR.  Weeks 5–8:  Duration will typically range from 40–60 minutes with an exercise intensity target from RPE 4–5 (“somewhat hard” to “hard”) and/or 50%–60% HRR. | Progression and/or regression will be prescribed based on how individual participants are tolerating their current exercise prescription.  If clinically appropriate and desired, participants may be prescribed greater than 150 minutes/week. For participants meeting exercise recommendations, exercise prescription will continue to include progressive overload principles (e.g., increased duration, intensity, speed, frequency).  To maintain intensity following physical adaptations to exercise, participants will be encouraged to increase the duration, speed, or load (e.g., incline) of their exercise sessions. Similarly, if disease or treatment burden reduces, exercise prescription will be progressed to maintain the target intensity. Conversely, if disease or treatment burden increases or exercise capacity decreases (for any reason) then exercise prescription will be adapted. |
| **Description of exercise (Item 8)** | |
| Aerobic exercise: any land-based exercise mode.  Aerobic exercise will target cardiovascular fitness, and participants will be provided with aerobic exercise prescription guidelines, individually tailored based on their preferences, baseline capacity, and goals. | Walking is preferred – but participants can choose any land-based exercise mode (e.g., cycling, jogging, exercise video). Aerobic modes that are not land-based (e.g., swimming) will be excluded from remotely monitored sessions as these cannot be remotely monitored using our platform. However, participants can complete these modes in non-supervised sessions.  Participants may be advised against certain aerobic exercise modes based on clinical judgement of safety, specificity for achieving goals, and participant mode of preference to maintain engagement. |
| **Home program component (Item 9)** | |
| If appropriate, participants will be encouraged to supplement remotely monitored exercise sessions with self-directed exercise on ≥2 other days. If aerobic, these sessions may be recorded through the REMOTE-COR-B platform. This may not be appropriate or achievable for some participants during the intervention period. | Written resistance training resources will also be provided to encourage participants to incorporate two sessions of resistance training per week into their exercise routine. |
| **Non-exercise components (Item 10)** | |
| Behaviour change education through direct messages. | See Item 6 for additional detail. |
| **Type and number of AE (Item 11)** | |
| Adverse event data will be regularly collected via comprehensive self-report and recorded following a trial-specific protocol. Participants will be asked to report any unfavourable or unintended sign, symptom, or disease temporally associated with the use of the intervention or outcome assessments, whether or not related to the intervention or outcome assessments.  Exercise professionals will prompt participants to report this information at each exercise testing session and during each remotely monitored exercise session (supported by an in-app function), as well as to report any adverse events that occurred during unsupervised exercise. Participants can also report adverse events at post-intervention and follow-up survey assessment. | Adverse events will be assessed based on frequency and severity, reported according to the Common Terminology Criteria for Adverse Events Version 5.0 grading system and the National Health and Medical Research Council guidelines for safety monitoring and reporting [32]. |
| **WHERE: location** |  |
| **Location (Item 12)** | |
| Intervention sessions will occur via the REMOTE-COR-B mobile app. | The platform utilises technology to facilitate remotely supervised exercise sessions anywhere the internet is available (e.g., at home, the gym, a park, or a favourite outdoor location). |
| **WHEN, HOW MUCH: dosage** | |
| **Dosage (Item 13)** | |
| 150 minutes per week at moderate intensity or above | Although exercise dosage (volume) is not the target of the intervention, exercise prescription will be tailored based on progressing participants towards this target, with consideration to individual characteristics.  A participant’s ability to achieve this target will depend on their individual circumstance and for some participants, this target may not be achievable during or following the intervention. Exercise professionals will encourage participants to do as much as is feasible within their personal circumstances. |
| **Frequency / Duration (Item 13)** | |
| 3 remotely monitored exercise sessions per week.  A minimum duration of 20 minutes per session, when possible, with a goal duration of 30-60 minutes. | Session duration will be tailored based on symptoms, prior medical history, exercise history, and intensity.  If 20 minutes of continuous exercise is not possible, participants will start by completing a shorter duration but at increased frequency (e.g., 2 x 10-minute bouts).  For complex cases/deconditioned participants interval training can be used to achieve goal duration. Interval training may also be used in fitter patients for variety. Otherwise, exercise will be continuous. |
| **Intensity (Item 13)** | |
| Exercise of at least moderate intensity, monitored via objectively measured heart rate (moderate: 40%HRR–60%HRR) or RPE using the Borg 0–10 Category-Ratio Scale (moderate: 3–5). | In the absence of HRR data, heart rate can be monitored as beats per minute. |
| **TAILORING: what, how** | |
| **Specificity (Item 14a)** | |
| The REMOTE-COR-B trial will place a strong emphasis on aerobic activity given the focus on cardiovascular health. The intervention will consist of aerobic exercise prescription on 3 days per week. |  |
| **Tailoring (Item 14b)** | |
| Individual components of the FITT principle (e.g., duration, intensity), will be prescribed and modified on an individual basis, with consideration to the goals, health status (e.g., side-effects, comorbidities), and exercise response of each participant. | Participants will be encouraged to meet the intervention target (3 remotely monitored sessions per week) and the recommended exercise dosage, through individualised exercise prescription that is appropriate to the needs, goals, and preferences of the participant. |
| **Starting level (Item 15)** | |
| Exercise prescription in Week 1 will be determined based on the participants cardiovascular fitness (as determined during baseline assessment), exercise history, comorbidities, treatment side-effects or disease symptoms, and participants goals. | The intensity of the first session will be guided by RPE to ensure that participants feel in control and to allow exercise professional to gain a sense of how RPE aligns with objective heart rate data. RPE can be requested throughout the exercise session. |
| **HOW WELL: planned, actual** | |
| **Fidelity (Item 16a)** | |
| Exercise professionals will attend a 2-hour training session covering the operation of the REMOTE-COR-B platform, including monitoring features, the live feedback function, and recording of exercise prescription and goals. In addition, exercise professionals will undertake practice sessions with other members of the research team.  After trial initiation, regular case discussions will be held between exercise professionals to discuss any issues or concerns and to ensure consistent intervention delivery between practitioners.  Implementation of the intervention will be monitored throughout the trial by the study coordinator, via auditing of exercise prescriptions and live coaching messages, and checks of average adherence rates. |  |
| **Planned Delivery (Item 16b)** | |
| The extent to which the intervention was delivered as planned will be reported through adherence data.  Detailed recording of changes to exercise prescription, symptoms, and adverse events will also occur within the REMOTE-COR-B platform, which will provide further information regarding the delivery of the intervention. Any intervention adjustments that may occur during the trial period (e.g., adjustment of set operating hours) will also be recorded. |  |

ACSM: American College of Sports Medicine; FITT: Frequency, Intensity, Time, Type; HRR: heart rate reserve; RPE: Rating of Perceived Exertion.
